# Stereotactic body radiotherapy for early-stage non-small cell lung cancer: clinical outcomes from a National Patient Registry

**DOI:** 10.1007/s13566-014-0177-0

**Published:** 2015-01-31

**Authors:** Joanne N. Davis, Clinton Medbery, Sanjeev Sharma, David Perry, John Pablo, David J. D’Ambrosio, Heidi McKellar, Frank C. Kimsey, Paul N. Chomiak, Anand Mahadevan

**Affiliations:** 1 The Radiosurgery Society, 1350 Dell, Suite 105, Campbell, CA 95008 USA; 2 Department of Radiation Oncology, St. Anthony Hospital, Oklahoma City, OK USA; 3 Department of Radiation Oncology, St. Mary’s Medical Center, Huntington, WV USA; 4 Department of Radiation Oncology, Medstar Franklin Square Medical Center, Baltimore, MD USA; 5 Department of Radiation Oncology, St. Joseph/Candler Hospital, Savannah, GA USA; 6 Department of Radiation Oncology, New Jersey CyberKnife at Community Medical Center, Toms River, NJ USA; 7 Department of Radiation Oncology, Eastern Texas Medical Center, Tyler, TX USA; 8 Department of Radiation Oncology, Erlanger Health System, Chattanooga, TN USA; 9 Thoracic Surgical Oncology, Sacred Heart Cancer Center, Pensacola, FL USA; 10 Department of Radiation Oncology, Beth Israel Deaconess Medical Center, Harvard Medical School, Boston, MA USA

**Keywords:** Stereotactic body radiotherapy, Lung cancer, Non-small cell lung cancer, Registry, Radiosurgery

## Abstract

**Objectives:**

Stereotactic body radiotherapy (SBRT) is a definitive local treatment option for patients with stage I non-small cell lung cancer (NSCLC) who are not surgical candidates and patients who refuse surgery. The purpose of this study was to assess the impact of SBRT on T1–T2 NSCLC from a national registry, reflecting practices and outcomes in a real-world setting.

**Methods:**

The RSSearch® Patient Registry was screened for T1–T2N0M0 NSCLC patients treated from May 2004 to May 2013 with SBRT. Descriptive analyses were used for patient, tumor, and treatment characteristics. Overall survival (OS) and local control (LC) were calculated using the Kaplan-Meier method.

**Results:**

In total, 723 patients with 517 T1 and 224 T2 lesions were treated with SBRT. Median follow-up was 12 months (1–87 months) with a median age of 76 years. Median SBRT dose was 54 Gy (range 10–80 Gy) delivered in a median of 3 fractions (range 1–5), and median biological equivalent dose (BED_10_) was 151.2 Gy (range 20–240 Gy). Median OS was 30 and 26 months for T1 and T2 tumors, respectively (*p* = 0.019). LC was associated with higher BED_10_ for T2 tumors, but not in T1 tumors at a median follow-up of 17 months. Seventeen-month LC for T2 tumors treated with BED_10_ < 105 Gy, BED_10_ 105-149, and BED_10_ ≥ 150 Gy was 43, 74, and 95 %, respectively (*p* = 0.011). Local failure rates for T2 tumors treated with BED_10_ < 105 Gy, 105–149 Gy, and ≥150 Gy were 32, 21, and 8 % (*p* = 0.029), respectively. Median OS for patients with T2 tumors treated with BED_10_ < 105 Gy was 17 vs. 32 months for T2 tumors treated with BED_10_ 105–149 Gy (*p* = 0.062).

**Conclusion:**

SBRT for T1–T2 NSCLC is feasible and effective in the community setting. OS was greater for patients with T1 lesions compared to T2 lesions. An improvement in LC was observed in patients with T2 lesions treated with BED_10_ > 105 Gy.

## Introduction

The standard treatment for patients with stage I (IA and IB) non-small cell lung cancer (NSCLC) with no medical contraindication is surgery. Surgery results in a loco-regional control rate of 90 % and a 5-year overall survival rate of 50–70 % for stage I NSCLC [1]. A significant number of early-stage lung cancer patients have co-morbidities which make them unsuitable for curative surgery. Radiation therapy is an alternative treatment for medically inoperable patients or patients who refuse surgery; however, conventional radiation therapy using 60 to 70 Gy results in relatively poor local control (30–70 %) and survival (15–30 %) [2, 3]. Stereotactic body radiotherapy (SBRT) is a form of ablative radiation therapy which delivers high doses of radiation in fewer fractions compared to conventional radiation therapy. At the same time, SBRT allows to minimize the dose to surrounding the normal lung, often diseased in this population, making them poor candidates for surgery in the first place. Studies show that SBRT improves local control rates of early-stage NSCLC with a local control rate of 85–98 % and 3-year overall survival rate of 48–65 % [4–13]. SBRT has been shown to reduce local recurrence in borderline surgical candidates with early-stage I NSCLC compared to limited resection [14–16]. SBRT is now considered a standard treatment for inoperable stage I NSCLC and is being explored as a treatment option for medically operable patients [17]. However, the optimal treatment and schedule of SBRT for T1–T2N0M0 lung cancer are still being explored.

Based on available evidence, technical capability exists to perform SBRT in the community setting and is being routinely done. As the role for SBRT to treat NSCLC expands in controlled prospective clinical trials, it will be important to understand how SBRT is being implemented in the community setting and to investigate the clinical outcomes. Patient registries can be powerful tools to describe treatment management patterns, understand variations in treatment and outcomes, study generalizability of clinical trial results, and can complement data from randomized clinical trials. Another potential benefit of a registry is that data for patient demographics, treatment practices, and outcomes can be captured from a large number of patients rapidly. The RSSearch® Patient Registry was designed to standardize data collection for patients treated with SRS and SBRT and currently includes screening, treatment, and outcome data for over 14,000 patients treated with SRS/SBRT [18]. The purpose of this study was to examine disease presentation, treatment practices, and clinical outcomes of patients with T1–T2N0M0 NSCLC enrolled in RSSearch®, thereby producing a real-world picture of disease, current treatment practices, and outcomes in radiation therapy using SBRT.

## Methods

A retrospective analysis of patients with histologically proven T1–T2N0M0 NSCLC treated with SBRT and enrolled in the RSSearch® Patient Registry (Clinicaltrials.gov Identifier:NCT01885299) was performed. The RSSearch® Patient Registry is managed by the Radiosurgery Society, a non-profit professional medical society. A description of the methodology, database design, and initial patient and treatment characteristics of patients enrolled in RSSearch® has been previously reported [18]. The database is housed by an independent third party, Advertek^SM^ (Louisville, KY), and meets all requirements to comply with the Health Insurance Portability and Accountability Act and Safe Harbor Policy to maintain system security, transmission of data, and patient confidentiality. All centers treating patients with SRS/SBRT clinically are offered and encouraged to participate in RSSearch®. Participation is voluntary, and no compensation is provided either to patients or participating centers. Each principal investigator is provided a copy of the RSSearch® Registry protocol, case report forms, sample patient informed consent, and web-based training for data entry and database navigation. Institutional Review Board (IRB) approval is required at all participating centers. All procedures followed were in accordance with the ethical standards of the responsible committee on human experimentation (institutional and national) and with the Helsinki Declaration of 1975, as revised in 2008 (5). All patients who are screened for potential SRS/SBRT treatment are eligible to be included in the RSSearch® Registry. Informed consent was obtained from all patients, as required by individual IRBs, prior to the patient’s data entered into the RSSearch® Registry. Retrospective analysis of RSSearch® is conducted from prospectively entered data. The selection of centers for this study included RSSearch® participating centers that treated NSCLC patients with SBRT between May 2004 and May 2013, with complete data entry fields for screening, treatment, and follow-up (minimum survival data) for their respective patients. For this analysis, NSCLC patients were treated at 14 institutions within the USA. Each center followed an independent Institutional Review Board (IRB)-approved protocol for RSSearch® participation.

Patients were treated with SBRT according to institutional guidelines. To compare the effects of various treatment protocols with different treatment fraction sizes and doses, the biological equivalent dose (BED) was calculated using the linear quadratic model, as BED = *D* × (1 + *d*/α/β) where *D* is the total dose, *d* is the dose per fraction, and the α/β ratio for the tumor was 10 Gy. Normal tissue dose restraints were reported by the treating institutions and captured in RSSearch® as the maximum point dose and interquartile range for each structure.

Patient follow-up was performed per institutional guidelines. All participating centers reported follow-up clinical and imaging data. Local control was evaluated independently for each lesion at the participating institution following a modified Response Evaluation and Criteria in Solid Tumors (RECIST) criteria. Lesion response was graded as either complete response (CR) defined as disappearance of all lesion/s treated, partial response (PR) defined as reduction in lesion size of lesions treated in by 30 % or greater, and stable disease (SD) defined as neither sufficient shrinkage nor sufficient increase of size of lesions. Local progression was defined as at least a 20 % increase in the size of lesions and/or appearance of one or more lesions in target treatment location and local control (LC) defined as disappearance of, decrease in, or no increase in size of the treated lesions. Analyses of LC, DC, and OS were calculated using the Kaplan-Meier method. LC was analyzed for each treated tumor whereas analysis of DC and OS was calculated for every patient. Specific cause of death was not reported for all patients in RSSearch® and therefore not evaluated in this study. Subgroups were compared using *X* ^2^ and log-rank statistics. Values of *p* < 0.05 were considered statistically significant. Statistical calculations were conducted using Instat and GraphPad Prism (La Jolla, CA).

## Results

### Demographics and lesion characteristics

In total, 723 patients with 741 lesions diagnosed with T1–T2 primary NSCLC lung cancer between May 2004 and May 2013 were included in this study. The median age was 76 years (range 41–95 years), 52 % were female and 48 % were male (Table 1); 88 % were Caucasian, 6 % African-American, and 0.7 % Hispanic. The median Karnofsky Performance Score was 80 (range 40–100). Three hundred seventy-four patients (52 %) were considered medically inoperable, 111 patients (15 %) were surgically inoperable, and the rest refused surgery. The primary co-morbidities for medically inoperable patients included pulmonary (*n* = 258), cardiac (*n* = 66), vascular (*n* = 9), and advanced age with poor performance status (*n* = 6). Eighty-four percent of patients had no prior treatment, 9 % had received chemotherapy, 2 % had undergone surgery, and 1 % had received external beam radiation therapy. Patients were most commonly referred to the radiation oncology department for SBRT evaluation by medical oncology (37 %), followed by pulmonology (21 %), cardio-thoracic surgery (17 %), other radiation oncology (10 %) departments, and self-referral (3 %). Medicare was listed as the primary payer for 72 % of patients, private insurance for 17 %, Medicaid for 2 %, Veterans Administration for 2 %, and uninsured/self-pay for 1 patient.

**Table 1:** Patient characteristics and demographics

Variable	Number
Gender: *n* = 723 patients	
Male	349 (48 %)
Female	374 (52 %)
Median age in years (range)	76 (41–95)
Median weight in lbs (range)	160 (60–330)
Median Karnofsky performance score (range)	80 (40–100)
Median VAS Pain Score (range)	0 (0–9)
Current or previous smoker	630 (87 %)
Median smoker pack/years (range)	50 (1–545)
Race/ethnicity	
Caucasian	634 (88 %)
Black/African American	42 (6 %)
Hispanic	5 (0.7 %)
Other	2 (0.3 %)
Unknown	39 (5 %)
Medically inoperable	374 (52 %)
Surgically inoperable	111 (15 %)
Prior Treatment(s): *n* = 741 lesions	
None	622 (84 %)
Chemotherapy	69 (9 %)
Surgery	17 (2 %)
External beam radiation	9 (1 %)
Radiosurgery	3 (0.4 %)
Radiofrequency ablation	3 (0.4 %)

The lesion characteristics are shown in Table 2. Seventy percent of lesions were T1 (*n* = 517) and 30 % were T2 (*n* = 224). The median lesion volume was 14.9 cc, and the median maximal tumor diameter was 2.4 cm (range 0.2–6.5 cm). Twenty-four patients had more than one lung tumor treated with SBRT (range 1–4 lesions). Pathological diagnosis was completed for all patients, and the histological subtypes were reported as adenocarcinoma (*n* = 248), NSCLC (*n* = 238), squamous cell carcinoma (*n* = 224), bronchiolo-alveolar carcinoma (*n* = 15), and large cell carcinoma (*n* = 16).

**Table 2:** Lesion and SBRT treatment characteristics

Variable	Number
T1N0M0	517 (70 %)
T2N0M0	224 (30 %)
Histology:	
Adenocarcinoma	248 (33 %)
Non-small cell carcinoma	238 (32 %)
Squamous cell carcinoma	224 (30 %)
Large cell carcinoma	16 (2 %)
Bronchiolo-alveolar carcinoma	15 (2 %)
Median lesion volume, cc (range)	14.9 (0.5–334)
Median lesion size, long axis, cm (range)	2.4 (0.2–6.5)
Lesion location	
Right upper lobe	197 (27 %)
Right middle lobe	38 (5 %)
Right lower lobe	116 (16 %)
Right lobe, NOS	5 (2 %)
Left upper lobe	185 (25 %)
Left middle lobe	13 (2 %)
Left lower lobe	94 (13 %)
Left lobe, NOS	9 (1 %)
Bronchus	3 (0.4 %)
Not indicated	81 (11 %)
Median SBRT dose (range), Gy	54 (10–80)
Median number of fractions	3 (1–5)
Median BED_10_ dose, Gy	151.2 (2–240)
Most common dose/fraction schemes:	
20 Gy × 3 = 60 Gy	219 (30 %)
18 Gy × 3 = 54 Gy	162 (22 %)
10 Gy × 5 = 50 Gy	79 (11 %)
12.5 Gy × 4 = 50 Gy	78 (11 %)
12 Gy × 4 = 48 Gy	45 (6 %)
12 Gy × 5 = 60 Gy	25 (3 %)
Median Dmax (range), Gy	72.7 (10–113)
Median monitor units	39,373 (5614–102,565)

### SBRT treatment

SBRT was indicated as the primary treatment for 91 % (*n* = 675) of lesions and adjuvant treatment for 8 % (*n* = 61) lesions. Treatment indication was not reported for five patients. The most common SBRT dose-fractionation schedules are shown in Table 2 and include 60 Gy in 3 fractions (*n* = 219), 54 Gy in 3 fractions (*n* = 162), 50 Gy in 5 fractions (*n* = 79), 50 Gy in 4 fractions (*n* = 78), and 48 Gy in 4 fractions (*n* = 45). The median dose was 54 Gy delivered in 3 fractions, and the median BED_10_ was 151.2 Gy (range, 20–240 Gy). The median maximum point dose (Dmax) to the target was 72.7 Gy (range 10–113 Gy).

Doses to organs at risk (OAR) were reported in RSSearch® as the Dmax for each individual OAR. Table 3 shows the median Dmax (range, 25th percentile and 75th percentile) for the esophagus, brachial plexus, trachea, main bronchus, heart, major vessels, and spinal cord. The median Dmax for esophagus, brachial plexus, trachea, main bronchus, heart, major vessels, and spinal cord were 9.9, 18.6, 13.5, 14.5, 12.5, 18.3, and 7.2 Gy, respectively. For comparison, the dose tolerance limits are also shown for Radiation Therapy Oncology Group (RTOG) 0236 protocol for medically inoperable patients treated with 54 Gy delivered in 3 fractions [7]. For all OARs, the dose reported at the 75th percentile was below the maximum dose limits specified in RTOG 0236.

**Table 3:** Doses to normal adjacent structures

Organ	Dmax, 25th Percentile (Gy)	Median Dmax (range)	Dmax, 75th Percentile (Gy)	Max dose constraints, RTOG 0236 [7]
Esophagus	6	9.9 Gy (0–51.1)	16	27 Gy max
Brachial plexus	4	18.6 Gy (0.02–43.5)	23.5	24 Gy max
Trachea	7	13.5 Gy (0–64.9)	26	30 Gy max
Main bronchus	8	14.5 Gy (0–63.0)	28	30 Gy max
Heart	7	12.5 Gy (0–96)	20	30 Gy max
Major vessels	10	18.3 (0.1–64)	34	39 Gy
Spinal cord	4.5	7.2 (0–75)	11.8	18 Gy

### Overall survival (OS), local control (LC), and distant disease control (DC)

**Fig. 1:**
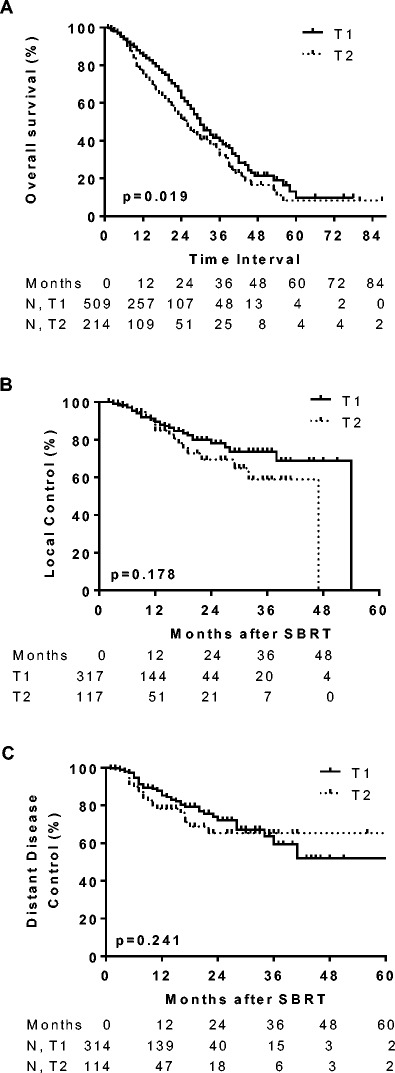
Survival curves for T1 and T2 lesions treated with SBRT. Kaplan-Meier analysis of overall survival (**a**), local control (**b**), and distant disease control (**c**) for patients with T1 (*solid lines*) and T2 (*dotted lines*) NSCLC treated with SBRT. *Ticked marks* indicate censored patients. Patients with T1 lesions had greater overall survival compared to T2 lesions (*p* = 0.019 by log-rank test)

The median follow-up was 12 months (range 1–87 months). Kaplan-Meier curves for survival are shown in Fig. 1. The median OS for the entire group was 29 months (95 % CI, 23–34 months). The median OS of patients with T1 and T2 tumors was 30 and 26 months, respectively (*p* = 0.019; Fig. 1a). One-year OS was 85 % (95 % CI, 81–88 %) for T1 and 76 % (95 % CI, 68–81 %) for T2 tumors. The 2-year OS rate was 63 % (95 % CI, 56–69 %) for T1 and 52 % (95 % CI, 42–60 %) for T2 tumors.

Four hundred and thirty-four lesions from 429 patients were evaluated for tumor response. A reduction in tumor size was reported in 254 lesions (59 %) with a complete response reported in 131 lesions and partial response reported in 123 lesions. Stable disease was reported in 62 (14 %) lesions. Local failure was reported in 57 lesions (13 %). One-year LC was 88 % (95 % CI, 84–92 %) and 2-year LC was 76 % (95 % CI, 68–82 %). When stratified by T-classification, 1-year LC was 89 % (95 % CI, 84–93 %) for T1 and 85 % (95 % CI, 74–91 %) for T2 tumors (Fig. 1b). The median time to local failure was 54 and 47 months for T1 and T2 tumors, respectively (*p* = 0.178). Seventy-six patients were reported to have distant disease progression. One-year DC was 85 % (95 % CI, 80–89 %) and 78 % (95 % CI, 68–86 %) for T1 and T2 tumors, respectively (Fig. 1c).

**Fig. 2:**
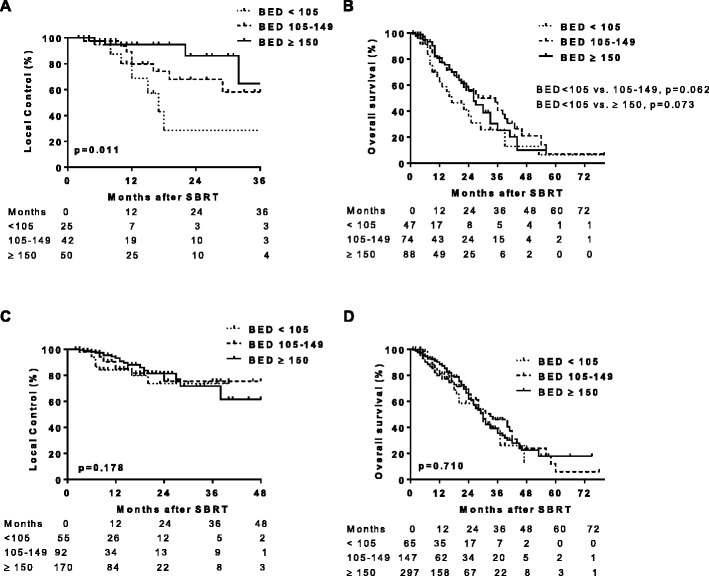
Local control (LC) and overall survival (OS) for T1 and T2 lesions treated with BED10 < 105 Gy vs. 105-149 Gy vs. ≥150 Gy. Kaplan-Meier analysis of LC for T2 (**a**) and T1 (**c**) lesions and OS for T2 (**b**) and T1 (**d**) lesions treated with BED10 < 105 Gy (*dotted line*), BED10 105–149 (*dashed line*), and BED10 ≥ 150 Gy (*solid line*). *Tick marks* indicate censored patients. LC improved for T2 lesions treated with BED10 (*p* = 0.011 by log-rank test), but not T1 lesions. There was a trend for improved OS for T2 lesions treated with BED10105–149 compared to BED10 < 105 although it did not reach statistical significance (*p* = 0.062 by log-rank test)

BED_10_ was calculated for each dose/fractionation regimen, and LC and OS were assessed for patients who received BED_10_ < 105 Gy vs. BED_10_ 105–149 Gy vs. BED_10_ ≥ 150 Gy. Local failure was associated with lower BED_10_ for T2 lesions. Local failure occurred in 32 % of patients treated with BED_10_ < 105 Gy vs. 21 % for BED_10_ 105–149 Gy vs. 8 % for BED_10_ ≥ 150 Gy (*p* = 0.029 by *X* ^*2*^ test; Table 4). Median time to local failure was 17 months for T2 tumors treated with BED_10_ < 105 Gy and not reached for BED_10_ ≥ 105. The 17-month LC rate was 43 % (95 % CI, 15–71 %) for BED_10_ < 105 Gy, 74 % (95 % CI, 50–88 %) for BED_10_ 105–149 Gy, and 95 % (95 % CI, 81–99 %) for BED_10_ ≥ 150 Gy (*p* = 0.011; Fig. 2a). There was no difference in local failure rates for T1 tumors treated with the different BED_10_ doses. Local failure was reported in 15 % of T1 lesions treated with BED10 < 105 Gy, 11 % for BED_10_ 105–149 Gy, and 11 % for BED_10_ ≥ 150 Gy (*p* = 0.713 by *X* ^*2*^; Table 4). The 1-year LC rate for T1 lesions was 84 % (95 % CI, 69–92 %) for BED_10_ < 105 Gy, 85 % (95 % CI, 71–93 %) for BED_10_ 105–149 Gy, and 93 % (95 % CI, 87–97 %) for BED_10_ ≥ 150 Gy (Fig. 2c).

**Table 4:** Local failures for T1 and T2 tumors stratified by BED_10_

T stage	BED < 105 Gy	BED 105–149 Gy	BED ≥ 150 Gy	*p* value (*X* ^2^ test)
T1	8/55 (15 %)	10/92 (11 %)	18/170 (11 %)	0.713
T2	8/25 (32 %)	9/42 (21 %)	4/50 (8 %)	0.029^a^

^a^Indicates statistically significant value

For patients with T2 lesions treated with BED_10_ > 105 Gy, there was a trend for improved OS, although it did not reach statistical significance. The 1-year OS rate for T2 lesions treated with BED_10_ < 105 Gy, BED_10_ 105–149 Gy, and BED_10_ ≥ 150 Gy was 63 % (95 % CI, 49–76 %), 78 % (95 % CI, 65–86 %), and 81 % (95 % CI, 69–88 %), respectively. Median OS was 17 vs. 28 months for BED_10_ < 105 Gy vs. BED_10_ 105–149 Gy (*p* = 0.062; Fig. 2b). There was no further improvement of OS for T2 lesions treated with BED10 ≥ 150 Gy compared to BED10 105–149 Gy (*p* = 0.62). Increasing BED_10_ was not associated with improved OS for T1 lesions. Median OS for patients with T1 lesions was 30, 33, and 30 months (*p* = 0.710) treated with BED_10_ < 105 Gy vs. BED_10_ 105 – 149 Gy vs. BED_10_ ≥ 150 Gy, respectively (Fig. 2d).

## Discussion

This study reports on the initial analysis of SBRT treatment of T1–T2 NSCLC from patients enrolled in the RSSearch® Patient Registry, the largest registry dedicated to SRS/SBRT treatment managed by a non-profit medical society. The goal of the current analysis was to evaluate the current management practices and outcomes of SBRT for early-stage lung cancer in a real-world setting. Our results demonstrate that participating centers are adhering to recommended treatment guidelines and published reports for SBRT treatment of T1–T2 lung cancer. The LC and OS rates reported in this study are in line with previous published reports of single institution, retrospective, and prospective studies [4, 5, 7–10, 12, 13, 19–21].

We acknowledge that this study is an observational study and that only randomized controlled clinical trials can conclusively determine survival benefits and differences in outcomes from treatment parameters; however, no such comparative efficacy or dose-escalation studies or data currently exist for SBRT for early-stage lung cancer. In the absence of this data, patient registries can provide important information to identify new treatment regimens, identify patients that may be most beneficial to treatments, generate new hypotheses on dose-response and response assessment, and thereby complement randomized clinical trials. In this study, we identified improved LC and improved OS in patients with T2 lesions treated with SBRT doses of BED_10_ > 105 Gy. Evidence exists for a dose-response relationship with standard fractionated radiotherapy and stage I NSCLC [22, 23]. Published results on dose-response relationships with SBRT and stages I–II NSCLC have been limited and controversial. Onishi et al. reported improved LC and OS in 257 patients with BED ≥ 100 Gy compared to BED < 100 Gy [6]. Grills et al. reported that BED_10_ predicted local relapse and distant metastasis in patients with T1–T3 N0M0 NSCLC with a 2-year local relapse of 15 % for BED_10_ < 105 Gy vs. 4 % for BED10 ≥ 105 [5]; however, BED_10_ dose relationships to local relapse were not stratified by T-classification in this study. In contrast, Stephans et al. did not find improvement for OS, LC, nodal failure, or distant metastasis in patients who received 60 Gy in 3 fractions (BED_10_ = 180 Gy) compared to patients who received 50 Gy in 5 fractions (BED_10_ = 100 Gy); however, the follow-up was limited with a median of 9.5 months (range 2.1–19.5 months) for patients (*n* = 38) that received 60 Gy in 3 fractions [24]. In our current study, the median time to local failure for T2 lesions with BED_10_ < 105 Gy was 17 months and not met for BED_10_ > 105 Gy, suggesting that follow-up longer than 17 months is needed to detect differences between BED < 105 vs. >105 Gy. Several studies using CyberKnife Robotic Radiosurgery System have reported a dose response for LC of early-stage lung cancer [21, 25, 26]. Le et al. reported 1-year LC rate of 91 % for patients treated with a single fraction of greater than 20 Gy vs. 54 % for patients who received a single fraction of less than 20 Gy [25]. Nuyttens et al. reported 2-year LC rate of 85 % for patients with centrally located tumors who received BED > 100 Gy (60 Gy delivered in 5 fractions) vs. 60 % for patients who received BED ≤ 100 Gy (45–50 Gy delivered in 5 fractions) [26]. van der Voort van Zyp et al. reported a 95 % LC for stage I patients who received 60 Gy delivered in 3 fractions vs. 78 % for patients who received 45 Gy delivered in 3 fractions [21]. In these studies, the effect of dose on T1 vs. T2 tumors was not studied. Onimaru et al. reported improved LC and OS in IB tumors treated to 48 Gy compared to 40 Gy delivered in four fractions, but did not find a response in IA lesions and suggested dose-response relationships may be related to tumor size [27].

There are several limitations of this study. First, as is common to registries, the patient inclusion criteria are not exclusively defined and the patient cohort represents a heterogeneous population. Our cohort included tumor sizes up to 6.5 cm, including an assortment of peripheral and centrally located tumors. Patients were also treated with a wide range of dose/fractionation schemes. Despite these variations, the LC rate in our study was 90 % and higher for T1 and T2 lesions treated with BED > 150 Gy and in line with previously published reports [5, 21, 25, 26, 28].

Another limitation of multi-institutional registries is obtaining complete follow-up information. While the majority of follow-up information was available, not all patients’ tumor response assessment was reported. Also, evaluation of recurrence did not require histological confirmation and relied on non-invasive tests including CT and PET. This is not uncommon in routine clinical practice. More importantly, this study did not report on toxicities consistently at this time and the causes of death are not always recorded. As this and other registries continue to mature, these aspects will be addressed and future studies on acute and late toxicities will be reported. Further long-term follow-up is warranted to determine whether the outcomes observed in this initial report will persist at 5 years and beyond.

Although SBRT for stage I lung cancer has been available for several years, there are continuous improvements in technology and expansion of indications for SBRT lung treatments. The RSSearch® Patient Registry continues to capture patient data, reflecting the treatment patterns and clinical outcomes from the real world. An advantage of patient registries is the ability to capture data on a large number of patients in a short time and report on outcomes rapidly. When information capture, including patient and treatment characteristics and follow-up data including response assessment and toxicity is done more rigorously, such studies could complement and validate results from randomized studies. Moreover, this may be a robust avenue to study applicability and generalizability of randomized clinical trials in the real-world community setting.

## Conclusions

We report on one of the largest multi-center datasets of early-stage NSCLC patients treated with SBRT and report on the current treatment management practices and outcomes in the community. Registries are useful tools to assess management practices and outcomes in the real world. Local control and survival outcomes for stage I NSCLC are not dissimilar to single institutional and prospective studies. Additionally, an improvement in LC was observed in patients with T2 lesions treated with BED_10_ > 105 Gy, supporting a dose response, which was not seen in patients with T1 lesions.
